# Association of Rad51 polymorphism with DNA repair in BRCA1 mutation carriers and sporadic breast cancer risk

**DOI:** 10.1186/1471-2407-11-278

**Published:** 2011-06-27

**Authors:** Luisel J Ricks-Santi, Lara E Sucheston, Yang Yang, Jo L Freudenheim, Claudine J Isaacs, Marc D Schwartz, Ramona G Dumitrescu, Catalin Marian, Jing Nie, Dominica Vito, Stephen B Edge, Peter G Shields

**Affiliations:** 1 Howard University Cancer Center, 2041 Georgia Ave, NW Washington, DC 20060, USA; 2 National Human Genome Center at Howard University, 2041 Georgia Ave, NW #615, Washington, DC 20059, USA; 3 Department of Biostatistics, University at Buffalo, State University of New York, Buffalo, NY 14214, USA; 4 Lombardi Comprehensive Cancer Center, Georgetown University Medical Cancer, 3800 Reservoir Rd, NW, Washington, DC 20057, USA; 5 Department of Social and Preventive Medicine, University at Buffalo, State University of New York, Buffalo, NY 14214, USA; 6 Department of Surgery, University at Buffalo, State University of New York, Buffalo, NY 14214, USA

## Abstract

**Background:**

Inter-individual variation in DNA repair capacity is thought to modulate breast cancer risk. The phenotypic mutagen sensitivity assay (MSA) measures DNA strand breaks in lymphocytes; women with familial and sporadic breast cancers have a higher mean number of breaks per cell (MBPC) then women without breast cancer. Here, we explore the relationships between the MSA and the *Rad51 *gene, which encodes a DNA repair enzyme that interacts with BRCA1 and BRCA2, in *BRCA1 *mutation carriers and women with sporadic breast cancer.

**Methods:**

Peripheral blood lymphoblasts from women with known *BRCA1 *mutations underwent the MSA (n = 138 among 20 families). *BRCA1 *and *Rad51 *genotyping and sequencing were performed to identify SNPs and haplotypes associated with the MSA. Positive associations from the study in high-risk families were subsequently examined in a population-based case-control study of breast cancer (n = 1170 cases and 2115 controls).

**Results:**

Breast cancer diagnosis was significantly associated with the MSA among women from *BRCA1 *families (OR = 3.2 95%CI: 1.5-6.7; p = 0.004). The *Rad51 *5'UTR 135 C>G genotype (OR = 3.64; 95% CI: 1.38, 9.54; p = 0.02), one *BRCA1 *haplotype (p = 0.03) and in a polygenic model, the E1038G and Q356R *BRCA1 *SNPs were significantly associated with MBPC (p = 0.009 and 0.002, respectively). The *Rad51 *5'UTR 135C genotype was not associated with breast cancer risk in the population-based study.

**Conclusions:**

Mutagen sensitivity might be a useful biomarker of penetrance among women with BRCA1 mutations because the MSA phenotype is partially explained by genetic variants in *BRCA1 *and *Rad51.*

## Background

The genetic determinants of breast cancer are under intensive study. Some women with a strong family history of breast cancer inherit *BRCA1 *or *BRCA2 *mutations, which have a variable penetrance for breast cancer, between 40 to 66% [1], suggesting that additional factors contribute to cancer risk among *BRCA1 *and *BRCA2 *carriers. For sporadic cancers, however, many low-penetrant single-nucleotide polymorphisms (SNPs) have been investigated in pathways ranging from growth factor signaling to DNA repair. Yet, it has been difficult to find consistency across study results [2-4], due to differences in study populations, sample sizes and study designs [5]. However, studies of high risk populations generally help uncover the molecular mechanisms of a disease and provide guidance and direction for studies of sporadic disease. While *BRCA1 *and *BRCA2 *mutations are highly penetrant [1], resulting in higher risk for breast cancer, both of these genes are also highly polymorphic. Moreover, several of their variants result in amino acid changes which could ultimately change the structure and function of the genes. It is therefore plausible that the combination of genetic changes in these genes or in genes in their pathway may at least contribute to the disease or the mechanisms associated with the disease in the general population. Another approach to enhancing the chances of identifying true positive associations is to conduct studies based on *a priori *hypotheses, for example by studying SNPs known to affect protein functions or levels. Thus, genotype-phenotype associations are needed to reduce the chances of false positive associations in breast cancer risk studies.

The mutagen sensitivity assay (MSA) provides a phenotypic marker of DNA repair capacity and genomic stress response, which has been reported as a heritable trait that affects both familial and sporadic breast cancer risk [6-9]. This assay measures the number of chromosomal breaks in cultured lymphocytes following exposure to DNA damaging agents. Different mutagens have been used, but gamma radiation has been the most widely utilized in breast cancer studies, because it is a direct DNA damaging agent whose effects are not dependent on cell penetration, metabolism, or clearance [10]. For example, using this assay, DNA from women in high-risk families and sporadic breast cancer cases exhibits about a 2-fold increase in the mean number of breaks per cell compared to DNA from women without cancer from low-risk families [6,7,11]. Thus, mutagen sensitivity may be a biomarker for DNA repair capacity and may specifically reflect differences in an individual's ability to repair DNA through the pathway of interest, homologous repair.

*BRCA1*, a nuclear protein that contains 24 exons (NC_000017.10), has a role in sensing DNA damage and cell cycle checkpoint control. It has been shown that *BRCA1*-deficient cells have DNA repair defects partially rescued by introducing exogenous wild-type *BRCA1 *[12], and that *BRCA1 *is a trigger of homology-directed DNA repair [12-14]. Many *BRCA1 *polymorphisms with allele frequencies >5% in Caucasians have been identified; however, only six of these (Q356R, D693N, P871L, E1038G, K1183R, and S1613G) result in amino acid changes (BIC-Breast Cancer Information Core; http://research.nhgri.nih.gov/bic/). These polymorphisms, with the exception of Q356R and D693N, are in significant linkage disequilibrium and are inherited as part of a shared haplotype [15]. Some studies have associated these SNPs with both familial and sporadic breast cancer risk, although there is a lack of consistency [16-24]. *BRCA1 *haplotypes have received some attention and haplotype-risk analysis has been done in several small and large case-control studies, but no associations between risk and haplotypes have been found [21,23,24]. Herein, we have chosen to study the *BRCA1 *polymorphisms Q356R, D693N, and E1038G because these SNPs could be functional variants associated with risk [25] and are in linkage disequilibrium with other SNPs of interest.

Rad51 was chosen for investigation because of their interactions with BRCA1 during homologous recombinational (HR) DNA repair [26]. Rad51 (RecA homolog, E. coli; NC_000015.9) has 10 exons that code for a 339 amino acid protein which forms a helical nucleoprotein filament on DNA [27]. Several studies of *Rad51 *among *BRCA1 *mutation carriers have found positive associations with cancer risk [28-32]. Cells deficient in BRCA1 are also defective in Rad51 irradiation-induced foci formation [26,33]. Experimental studies show that the loss of Rad51 may drive genetic instability, chromosomal aberrations, and carcinogenesis by facilitating an accumulation of genetic changes [34-36]. Rad51 is over-expressed in a *BRCA1 *mutant cell line and rescues cells from apoptosis [37]. Studies have reported that the *Rad51 5'UTR *variant 135C allele (rs1801320) was associated with a decreased risk of breast cancer in *BRCA1 *5382insC mutation carriers [29] and other mutation carriers [32], while no association was found in a case-control study of sporadic breast cancer [38,39]. Antoniou et al. reported that the SNP modified breast cancer risk among *BRCA2 *mutation carriers and *BRCA1 *loss-of-function mutation carriers [40]. Although the functional consequences of the 135G>C polymorphism is unknown, it is speculated that because it alters a CpG island in the promoter, it may regulate expression and affect mRNA levels [40,41]. Additionally, there is some evidence of an association between this variant and decreased Rad51 protein expression in *BRCA1/2 *mutation carriers [42]. Although, there have been some reports of *Rad51 *haplotypes associated risk in high-risk families [31,43,44] and with sporadic breast cancer risk [31,43,44], these haplotypes are composed only of SNPs in the Rad51 putative promoter, introns, or the 3' un-translated region. To date, there are no reported Rad51 haplotypes composed of SNPs in the coding region, indicating the coding region is well conserved [45].

In order to identify SNPs and haplotypes in *BRCA1*and *Rad51 *that might affect familial and sporadic breast cancer risk, we conducted a study of genotype-phenotype relationships. First, we compared and validated the mutagen sensitivity assay in Epstein Barr Virus (EBV)-immortalized lymphocyte cell lines, comparing the lymphoblast results to freshly cultured lymphocytes from whole blood. We, then, used the MSA to study associations between genotypes and haplotypes as they relate to DNA-repair capacity in EBV-immortalized lymphocytes from 138 women with known *BRCA1 *mutations. We then applied these results to a population-based case-control study of breast cancer.

## Methods

### Subjects

#### Familial Cancer Registry

Participants for this study were identified through the Lombardi Comprehensive Cancer Center (LCCC) Familial Cancer Registry (FCR). We included all FCR participants with known *BRCA1 *mutations and female family members who had EBV-immortalized lymphoblasts available for study. Notably, several of the female family members have tested negative (true negatives) for the familial mutation. However, because we were also interested in subsequent breast cancer risk, FCR participants who had undergone prophylactic mastectomy and oophorectomy were excluded from the study, although many subjects were still contemplating these procedures. This study received approval by the Institutional Review Board at Georgetown University.

#### Case-Control Study of Sporadic Cancer Risk

Subjects were recruited for the Western New York Exposures and Breast Cancer (WEB) study, a large, population-based case-control study conducted between 1996 and 2001 (n = 3285). This study has been described in detail elsewhere [46]. Cases (n = 1170) were women with incident breast cancer between the ages of 35 and 79 years from Erie and Niagara counties. Controls (n = 2115) were randomly selected from the same counties using lists of driver's license enrollees provided by the New York State Department of Motor Vehicles for those less than 65 years of age, and the Health Care Finance Administration for those 65 years of age and older. Controls were frequency matched by age and race to cases. The protocol was approved by the Institutional Review Board at the University at Buffalo and Georgetown University, as well as by the review boards of the participating hospitals. A detailed interviewer-administered questionnaire was used to assess the use of breast cancer risk factors.

### Lymphoblast preparation

FCR participants underwent phlebotomy, providing lymphocytes for EBV-immortalization using previously described methods [47,48]. Briefly, equal amounts of blood and phosphate buffered solution (PBS) (Mediatech, Inc, VA) were slowly added to a tube filled with ficol (Amersham Biosciences, Sweden) to obtain clear separation of blood components. The mixture was centrifuged at 400 g. The lymphocyte layer was removed and added to Epstein Barr virus (EBV) supernatant (ATCC, VA), Cyclosporin A (Biomol International LP, PA) and RPMI1640 medium supplemented with 10% fetal calf serum (Sigma, MO), 2% L-glutamine (GIBCO, CA), 1% Sodium Pyruvate (GIBCO, CA), 1% NEAA (non-essential amino acids-GIBCO, CA), 0.1% 2-mercaptoethanol (GIBCO, CA), and 0.1% gentamycin (Invitrogen, CA). After several media changes, 2-3 days apart, and incubation at 37°C in 5% CO_2_, cell pellets were transferred, kept in cell culture freezing media (GIBCO #11101-011) and stored in liquid nitrogen by Georgetown University's Tissue Culture Shared Resource.

### Mutagen Sensitivity Assay

The MSA was performed on EBV-immortalized lymphoblastoid cell lines from all 138 FCR participants and on fresh whole blood lymphocyte cultures from a subset of 19 women, in order to validate the use of the cell lines against cultured blood lymphocytes. Fresh whole blood, within 24 hours of collection, was incubated in RPMI1640 medium (GIBCO, CA) supplemented with 20% fetal calf serum (Sigma, MO) and phytohemagglutinin (GIBCO, CA) at 37°C for 67 hours in 5% CO_2_. The EBV-immortalized lymphoblastoid cells were cultured similarly, except the culture media was supplemented with fetal calf serum (10%; Sigma, MO), L-glutamine (2%; GIBCO, CA), sodium pyruvate (1%; GIBCO, CA), non-essential amino acids (1%; GIBCO, CA), 2-mercaptoethanol (0.1%; GIBCO, CA), and gentamycin (0.1%; Invitrogen, CA). After 67 hours, the cells were irradiated with 1 Gy gamma radiation (^137^Cs source gamma research irradiator), according to the method of Sanford and Parshad [49,50]. After further incubating for 4 hours, the cultures were treated with colcemid (0.04 ug/ml; GIBCO, CA) to arrest the cell cycle. The cells were then treated with hypotonic solution (0.06 mol/L KCl; Sigma, MO), fixed [3:1 methanol (Sigma, MO): glacial acetic acid (Fisher, PA)] and then metaphase spreads were established and Giemsa stained (Sigma-Aldrich Corp., MO). The frequency of chromatid breaks per cell (b/c) was calculated from metaphase spreads as a measure of an individual's DNA repair efficiency. Fifty well-spread, clear, and complete metaphases per culture were scored, and then the mean number of breaks per cell (MBPC) was determined for each subject. Readings were blinded to subject, cancer status, replicate and paired-sample status.

For validation purposes, MSA comparisons were made on corresponding fresh peripheral blood and EBV-immortalized lymphocytes for 19 participants from the FCR. Intra-individual variation of MBPC between concordant fresh blood and immortalized lymphocytes was assessed using the coefficient of variation (CV = SD/μ).

### DNA Sequencing

*BRCA1 *direct sequencing was done by Myriad Genetics, Inc. (Salt Lake City, UT). For *RAD51 *exon sequencing, genomic DNA was extracted from the EBV-immortalized lymphoblastoid cell culture pellets using a Qiagen M48 Biorobot (Qiagen, # 9000708) and the MagAttract^® ^DNA Mini M48 Kit (Qiagen, #953336), or by phenol-chloroform-isoamyl alcohol methods [51]. PCR was first performed using DNA (5-10 ng), AmpliTaq Gold^® ^PCR Master mix (2X; Applied Biosystems #58004012-01, Foster City, CA), glycerol (50%; Sigma, MO) and VariantSEQr ™ RSA primer mix (Applied Biosystems, Foster City, and CA). The reaction was run on the GeneAmp^® ^PCR system 9700 and the thermal cycler program was as follows: 96°C for 5 minutes; 40 cycles of 94°C for 30 seconds, 60°C for 45 seconds and 72°C for 45 seconds and extension at 72°C for 10 minutes. The PCR product (5 ul) was treated with ExoSAP-IT (Exonuclease I and Shrimp Alkaline Phosphatase-USB 7820) to degrade unused reagents. For the sequencing reaction, PCR product (10-20 ug; 1-2 ul final PCR volume), M13 Universal Forward Primer (5 pmole/μl; 5'TGTAAAACGACGGCCAGT-3') and DYEnamic ET terminator reagent premix (Amersham Biosciences, Piscataway, NJ) were subjected to PCR (30 cycles of 95°C, 20 s; 50°C, 15 s; 60°C, 1 min). For the post reaction clean-up, an AutoSeq96 plate (Amersham Biosciences, Piscataway, NJ) was used. Sequencing was performed with a capillary sequencer (MegaBACE 1000, GE Healthcare Bio-Sciences Corp., Piscataway, NJ) and the data were analyzed with the Sequencher software (Sequencher 4.7, Gene Codes Corporation, Ann Arbor, MI). Twenty percent of the sequences were repeated for quality control and mutations were confirmed by running PCR products in reverse sense with M13 Universal reverse primer (5'CAGGAAACAGCTATGACC-3'). Subjects in the highest and lowest quartiles of MBPC for the MSA were chosen for *Rad51 *sequencing of the coding regions (n = 92).

### Genotyping and Haplotyping

For the FCR subjects, we genotyped three SNPs in *BRCA1*, namely D693N (rs4986850), Q356R (rs1799950), and E1038G (rs16941). One SNP in *Rad51 *was genotyped, namely 5'UTR 135G>C (rs1801320). Genotyping was carried by allelic discrimination Real Time PCR with TaqMan probes using primers and probes from Applied Biosystems (Applied Biosystems, Foster City, CA) as previously described [52]. Briefly, TaqMan^® ^Universal PCR Master Mix (Applied Biosystems, Foster City, CA) and TaqMan^® ^SNP Genotyping Assay Mix were combined with 5-10 ng of genomic DNA. PCR was conducted using the ABI Prism 7900HT Real Time PCR instrument (Applied Biosystems, Foster City, CA) with the following amplification protocol: 50°C, 2 minutes; 95°C, 10 min and 49 cycles of 92°C, 15 s and 60°C, 1 min. HapMap genotype data for Caucasians [15] and Haploview^© ^(Haploview 3.32, Broad Institute of MIT and Harvard, Boston, MA) [53,54] were used to identify *BRCA1 *tag SNPs. Input files of LCCC-FCR *BRCA1 *genotyping results were also used to identify tag SNPs. The genotyping techniques and methods mentioned above where also used for tagging SNPs with probes and primers from Applied Biosystems using the same PCR conditions.

### Statistical Analysis

#### MSA Assay

To compare MSA MBPCs in whole blood culture and EBV cell lines, the Spearman rank correlation statistics (rho) was calculated. The Wilcoxon signed rank test was also performed to compare the MBPC from whole blood to that of EBV-transformed cell lines. These analyses were done with SPSS (version 12.0 for windows).

#### Genotype-Phenotype Association Analysis in FCR cohort

Because the FCR cohort includes related individuals, two association analyses were performed; we analyzed the entire cohort, taking into account familial relationships, and we also analyzed only unrelated probands or the first affected family member who sought medical attention (n = 110).

For the family-based analysis, SAGE (Statistical Analysis for Genetic Epidemiology, Release 6.0.1) [55] was used to calculate familial correlations (FCOR) (e.g., parent-offspring and sibling), which were then compared for MBPC. The relationships analyzed were sister: sister (n = 15), aunt-through-mother: niece (n = 6), female-cousin-through-father: female-cousin-through-father (n = 3), and female-cousin-through-mother: female-cousin-through-mother (n = 7). A variance-component model developed for family-based association was used to assess single SNP association with the continuous measure of MBPC [56-58]. The model used for analysis of MBPC is as follows:

where *i *is the individual or a pair (i.e. sister pair, aunt through mom: niece, etc.), *z*_*i *_is a genotype indicator with effect coding, *h *is the generalized modulus power transformation [59], *p*_*i *_is a random polygenic effect, the c_i _are covariates, and *ε*_*i *_is a random residual individual effect.

Analysis of unrelated individuals was done by dichotomizing subjects as having high or low MBPC, based on the median value in unaffected subjects (median = 0.22 MBPC). Chi-square tests for independence were performed to assess the relationship between MBPC and genotypes. Fisher's exact test was used for 2 × 2 tables when cells had a frequency lower than 5. The association between genotypes/haplotypes and MBPC was examined with logistic regression using genetic modeling (co-dominant, dominant, and log-additive models), and odds ratios with 95% confidence intervals were calculated, adjusting for age and stratifying by *BRCA1 *mutation.

#### Haplotype Analysis

SNP allele frequencies in unrelated affected and unaffected subjects were tested for Hardy-Weinberg equilibrium (Graphpad Software) [60]. Haplotypes were constructed using the PHASE software program (PHASE 2.1, Department of Statistics, University of Washington, Seattle, WA) [61,62]. To assess the sensitivity of the regression results to the uncertainty in the estimated haplotypes, we simulated 10 datasets utilizing the haplotype probabilities generated by PHASE.

#### Case-control analysis

Statistical analyses of the case-control study were done with the SAS/STAT^® ^software (version 9.1, SAS Institute Inc., Cary, NC). Allele frequencies in cases and controls were tested for Hardy-Weinberg equilibrium. The associations for disease status and polymorphisms were analyzed using logistic regression. Odds ratios and 95% confidence intervals were adjusted only for age and first-degree relative with breast cancer. Two-sided *p-*values of ≤ 0.05 were considered as statistically significant. Using the Bonferroni test, p-values were adjusted for multiple testing (p = 0.013). For *Rad51 *5'UTR 135G>C genotyping, the GC and CC genotypes were combined for regression analysis due to the low proportion of subjects with the CC genotype (<1%).

## Results

### Demographics

Table 1 provides the demographic information for subjects selected from the FCR. In total 138 eligible women were identified, 73 women with known *BRCA1 *mutations and a history of breast cancer (affected), 3 affected *BRCA1 *negative, 6 affected *Jewish panel *negative (185delAG, 5382insC), 48 women with known *BRCA1 *mutations and no history of breast cancer (unaffected) and 8 women negative for the mutation found in the family member (true negative), for a total of 56 unaffected subjects. Sixty two of the participants were related (from a total of 20 families). The mean age of the unaffected participants was 47.44 (SD = 13.63; range = 25-78) and the mean age of the affected participants was 44.73 (SD = 10.65; range = 27-79).

**Table 1:** Demographics of high-risk breast cancer subjects

	Unaffected	Affected	p-value
	*N = 56*	*N = 82*	

**Age at Diagnosis Range (yrs)**	25-78	27-79	

**Mean Age at Diagnosis (yrs)**	47.44	44.73	0.19*

**Median Age at Diagnosis (yrs)**	50	45	

**Standard Deviation**	13.63	10.65	

***Mutation***			

*185delAG (BRCA1)*	19 (31.6%)	21 (24.7%)	

5382insC	4 (7.0%)	12 (14.8%)	0.15**

Other	35 (61.4%)	49 (60.5%)	0.57**

	**Relationship Information**		

		Num. Pairs	

Sister-Sister		15	

Other relationship			

i.e. Aunt-Niece and Cousins		16	

*Unpaired t-test, t = 1.13, df = 136

**Determined by Fisher's exact test.

### Mutagen Sensitivity Assay Validation

**Figure 1:**
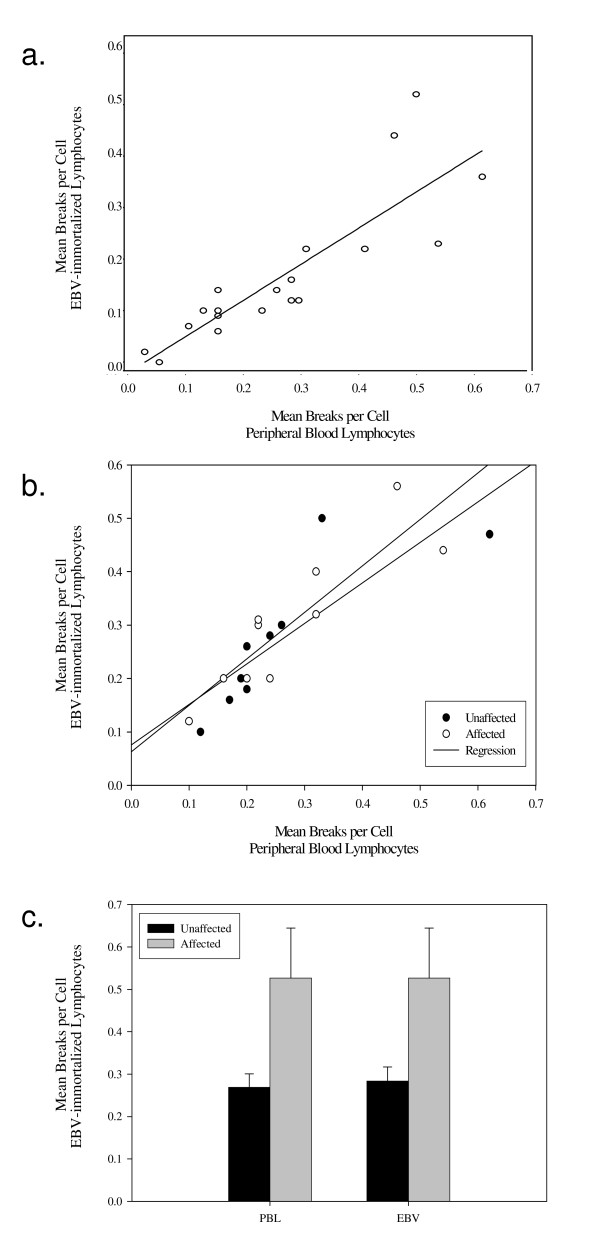
**Correlation of MBPC in peripheral blood lymphocytes and EBV-immortalized cell lines**. a) Scatter plot of paired peripheral blood lymphocytes and EBV-immortalized cell lines MBPC. b) Peripheral blood lymphocytes and EBV-immortalized cell line MBPC stratified by cancer status. c) Bar graph representing stratification by cancer status and type of cell line.

Comparisons were made on corresponding fresh peripheral blood and EBV-immortalized lymphocytes using the MSA assay for 19 participants from the FCR. The MBPC (mean number of breaks per cell) was 0.27 (SD = 0.14) in fresh blood and 0.29 (SD = 0.13) in EBV-immortalized cell lines (difference = -0.021 ± 0.073; p = 0.17). The MBPC in EBV-immortalized lymphoblastoid cell lines was highly correlated with the MBPC in freshly cultured PHA lymphocyte-stimulated whole blood from the same subjects (Figure 1a.; rho = 0.92, 95%CI = 0.79-0.97). Intra-individual variation of MBPC between concordant fresh blood and immortalized lymphocytes was assessed using the CV and ranged from 0-28.9% (mean CV = 13.0%). An analysis was done separately for affected and unaffected individuals, and although the sample sizes were small, results were similar to the pooled analysis (Figures 1b and 1c).

### MBPC correlations between relative pairs

Analyses revealed that MBPC among the 15 sister pairs were poorly correlated (r = 0.27) and not statistically significant (p = 0.33). None of the other relationships, aunt-through-mother: niece, female-cousin-through-father: female-cousin-through-father, and female-cousin-through-mother: female-cousin-through-mother showed correlations significantly different from 0 at the 0.05 level either (r = 0.16 ± 0.19 [p = 0.41], -0.98 ± -0.28 [p = 0.06], -0.44 ± -0.22 [p = 0.08], respectively).

### MBPC in affected and unaffected *BRCA1 *carriers

In the family-based analysis using all 138 participants and adjusting for familial correlation and dichotomizing subjects as low or high MBPC, we found an association between MBPC and breast cancer status (OR = 3.2 95%CI: 1.5-6.7; p = 0.004). The variance component-model also showed a correlation between MBPC and breast cancer diagnosis (p = 0.004). Analysis of unrelated individuals, also after dichotomizing MBPC, showed an increased risk that was not statistically significant (n = 110, OR = 1.6, 95% CI: 0.7-3.7; p = 0.34). Additionally, when unrelated affecteds (n = 76) were compared to unrelated true negatives (n = 8), true negatives had lower MBPC, and the results just failed to reach statistical significance (p = 0.07). A summary of MBPC statistics in affected and unaffected can also be found in additional file 1.

### *Rad51 *Sequencing Results

Exons 2-9 were sequenced (n = 92) and analyzed in subjects in the upper and lower quartiles of the distribution for MBPC (n = 79). Although several genetic variants were discovered and confirmed among subjects, none had a frequency >5% and were thus not pursued for genotyping and haplotyping.

### Genetic Associations

The *BRCA1 *E1038G, D693N, Q356R, and *Rad51 *5'UTR 135G>C genotypes followed Hardy-Weinberg equilibrium in the unaffected subjects (*p *= 0.70, 0.61, 0.29, and 0.39, respectively). There was no statistically significant association using any of the genetic models for the *BRCA1 *E1038G, D693N, and Q356R genotypes and MBPC either among unrelated individuals (n = 110) (Table 2) or among all subjects as assessed by the variance component model. However, in an analysis of family members only, the polygenic model revealed that the E1038G and Q356R *BRCA1 *SNPs were significantly associated with MBPC (p = 0.009 and 0.002, respectively). BRCA1 mutation-SNP interactions for associations with MBPC were also examined via stratification by status for BRCA1 mutation. For 185delAG and 5382insC mutation carriers, there were no associations between *BRCA1 *genotypes and MBPC in unrelated subjects after adjusting for age (Table 3).

**Table 2:** Relationship between mutagen sensitivity and polymorphisms in unrelated subjects (n = 110)

		Genetic Model	Genotype	Low		High		OR*	95% CI	p
BRCA1				n = 44	%	n = 66	%			

	E1038R	Co-Dominant	TT	20	18.2%	23	20.9%	1.00		

			CT	18	16.4%	32	29.1%	1.08	0.45 - 2.64	

			CC	6	5.5%	11	10.0%	1.07	0.32 - 3.60	0.46

		Dominant	CC/CT	24	21.8%	43	39.1%	1.60	0.47 - 2.47	0.34

		Log-Additive							0.64 - 1.94	0.70

	D693N	Dominant	CC	41	37.3%	57	51.8%	1.00		

			CT	1	0.9%	9	8.2%	6.03	0.69 - 52.02	0.10

	Q356R	Dominant	TT	32	29.1%	56	50.9%	1.00		

			CT	11	10.0%	9	8.2%	0.57	0.20 - 1.60	0.28

Rad51	5' UTR 135G>C									

		Dominant	GG	36	35.0%	35	34.0%	1.00		

			CC/CG	7	6.8%	25	24.3%	3.64	1.38 - 9.54	0.03

*Adjusted for age

**Table 3:** Relationship between mutagen sensitivity and polymorphisms in BRCA1 185delAG and 5382insC mutations carriers who are unrelated

		Genotype	Low		High		Fisher's p
185delAG			n = 8	%	n = 17	%	

	E1038G	TT	0	0	0	0	

		CT	3	20.0%	8	53.3%	

		CC	3	20.0%	2	13.3%	0.25

	D693N	CC	4	28.6%	8	57.1%	

		CT	0	0.0%	2	14.3%	>0.99

	Q356R	TT	5	17.9%	7	25.0%	

		CT	1	3.6%	1	3.6%	0.45

	Rad51	GG	8	32.0%	10	40.0%	

		CG/CC	0	0.0%	7	28.0%	0.06

							

			Low		High		Fisher's p

5382insC			n = 3	%	n = 8	%	

	E1038G	TT	2	28.6%	3	42.9%	

		CT/CC	0	0.0%	2	28.6%	>0.99

	D693N	CC	2	28.6%	4	57.1%	

		CT	0	0.0%	1	14.3%	0.73

An association with MBPC was found with the *Rad51 *5'UTR 135 CC/CG genotypes; the combined homozygous CC genotype and the heterozygote had an age-adjusted OR of 3.64 (95% CI: 1.38-9.54; p = 0.03) for unrelated women (Table 2). However, after adjusting for multiple testing using the Bonferroni test, this SNP did not remain significantly associated with MBPC. Nevertheless, the variance-component model also revealed a significant association between MBPC and the *Rad51 *5'UTR 135 SNP (p = 0.02). In unrelated individuals, subjects carrying both the 185delAG mutation and the *Rad51 *5'UTR C allele, also, tended to have higher MBPC (p = 0.06) (Table 3).

### Haplotype results in high-risk subjects

Myriad Genetics, Inc. (Salt Lake City, UT) sequencing data for 35 individuals were used to construct *BRCA1 *haplotypes. The tagging SNPs identified by the Haploview software revealed that *BRCA1 *E1038G, Q356R, and D693N tagging SNPs could identify 5 haplotypes in Caucasians, totaling 95% of possible haplotypes. After genotyping the aforementioned tag SNPs in all of the probands (n = 110), *BRCA1 *haplotypes were reconstructed. Linear regression models indicated that BRCA1 haplotype, CTC, yields a lower risk of high MBPC after adjusting for age (p = 0.03) (Table 4). However, this haplotype was only present in approximately 3.8% of subjects. In 4 out of 10 simulated datasets, this haplotype had p ≤ 0.03 with non-significant results being associated with a haplotype frequency ≤3. Haplotype analysis of *Rad51 *was not performed because sequencing analysis did not reveal SNPs with >5% frequency in the coding/exonic region.

**Table 4:** Linear Regression of MBPC on *BRCA1 *Haplotypes in Unrelated Individuals

Halpotype	Frequency	%	PARAMETER	P-value	PARAMETER*	P-value*
C C T	9	6.8%	0.10	0.74	0.09	0.75

C T C	5	3.8%	-0.80	**0.03**	-0.81	0.03

T C C	41	31.1%	0.08	0.68	0.08	0.68

T C T	67	50.8%	0.28	0.20	0.28	0.20

T T C	9	6.8%	-0.02	0.94	-0.02	0.95

T T T	1	0.8%	-1.47	0.07	-1.48	0.07

*Adjusted for Age.

† First Position = Q356R; second position = D693N; third position = E1038G

### Analysis of Rad51 in the case-control study of sporadic breast cancer

In the control population, the *Rad51 *5'UTR 135G>C genotypes were in Hardy-Weinberg equilibrium proportions in self-reported white women (p = 0.30), but not in women who self-reported as non-white (p = 0.07). Genotype distributions in whites (n = 2741) and non-whites (n = 253) were significantly different (χ^2 ^= 87.32, p < 0.001), and because of the violation of Hardy-Weinberg equilibrium proportions and significant allele frequency difference, all subsequent analyses were performed using the self-reported white population only.

There was no association of the *Rad51 *5'UTR 135G>C genotype with breast cancer risk in either pre- or postmenopausal women. Adjusted and unadjusted models were similar and adjusting did not substantially change the estimation of the OR. Specifically, adjusted ORs were 0.87 (95% CI 0.57-1.31) and 1.11 (95% CI 0.86-1.44) for pre- and postmenopausal women, respectively (Table 5). Characteristics of WEB participants by case-control status and Rad51 SNP can be found in additional file 2.

**Table 5:** Risk of Breast Cancer by *Rad51 *5'UTR 135G>C among whites, WEB study

	Cases	Controls	Crude OR (CI)	Adjusted OR* (CI)
Pre-menopausal				

GG	236	439	1.00	1.00

CG + CC	43	86	0.93 (0.62-1.39)	0.87 (0.57-1.31)

Post-menopausal				

GG	594	1036	1.00	1.00

CG + CC	122	185	1.15 (0.90-1.48)	1.11 (0.86-1.44)

*Odds ratios and 95% confidence intervals adjusted for age and first-degree relative with breast cancer.

## Discussion

In this study, we found an association between the MSA and breast cancer, and some genotype-phenotype relationships, in subjects from high risk breast cancer families. While there was no overall association for the MSA with *BRCA1 *Q356R, D693N, and E1038G genotypes in unrelated individuals, associations were found among family members using the polygenic model where the E1038G and Q356R *BRCA1 *SNPs were significantly associated with MBPC. Furthermore, the rare CTC (356R, 693N, and 1038G) haplotype also was found to be associated with the MSA. As for *Rad51*, those with the 5'UTR 135C SNP had statistically significantly higher MBPC than those who had the wild-type allele. When stratified by 185delAG or 5382insC mutation carriers, only 185delAG carriers with the *Rad51 *5'UTR 135C allele were marginally associated with higher MBPC. On the other hand, the *Rad51 *SNP was not associated with risk in a population-based study of sporadic breast cancer. These data indicate that mutagen sensitivity, and therefore, DNA repair capacity, might be a useful biomarker for determining penetrance among women with *BRCA1 *mutations, and that the mutagen sensitivity phenotype is partially explained by genetic variants in *BRCA1 *and *Rad51.*

The MSA, as a phenotypic assay, has been generally applied to freshly collected peripheral blood lymphocytes. Because we aimed to identify SNPs in genotype-phenotype relationships from EBV-immortalized studies and relate them to breast cancer risk, we first assessed the correlation of MSA results between EBV-immortalized and fresh peripheral blood lymphocytes. Since EBV-immortalized lymphoblasts are primarily derived from B-lymphocytes, while PHA-stimulated whole blood cultures yield primarily T-lymphocytes, it was possible that these lymphocyte subpopulations would yield quantitative differences in MBPC, and that the classification of women by high and low MBPC could be different. In this report, we demonstrate that the results for both assays were statistically related and quantitatively similar.

Mutagen sensitivity is regarded as a heritable trait as reviewed by Wu et al [63]. Given that our sample included several families with women of differing relationships, the correlation of MBPC between family members was calculated and the sibling (sister:sister) correlation was found to be consistent with previous reports of mutagen sensitivity in dizygotic twins; the correlation coefficient for sisters in this study (r = 0.33), albeit not statistically significant due to a small sample size, was very similar to the study by Wu et al. (r = 0.27) [64].

In this study, MBPC was significantly higher in the group of affected cases compared to women without cancer when the variance-component model developed for family-based association was applied [56-58]. However, when related cases were removed from the analysis, the OR remained elevated but was not statistically significant. It may be that including related cases biased the results because the MSA is a heritable trait (although only with a correlation of 0.33), or that removing the related subjects resulted in lower statistical power due to a smaller sample size. Although the study has a small number of families and the pedigrees are sparse, the variance-component model is considered more informative than the unrelated family member analysis because of its ability to simultaneously estimate residual and multi-factorial (polygenic, familial, marital or sibling) variance components. In addition this approach uses the quantitative trait as is without dichotomizing, thus possibly increasing the power to correctly detect allelic association. Also, the method combines the original association method by George and Elston, with the pedigree TDT-type analysis in such a way as to maximize power [58,65]. Although familial components can be incorporated into the equation, the random polygenic effect was the only variance component included when analyzing the two populations. Moreover, when unrelated affecteds were compared to true negatives (unrelated unaffected women without the BRCA1 mutation), the difference was borderline statistically significant (p = 0.07), further supporting a true relationship. Our results are consistent with other studies showing decreased DNA repair capacity in both comparisons of breast cancer cases to controls from population studies and from studies of high risk families [6-9]. However, differences in findings could also reflect how DNA repair capacity was measured.

*BRCA1 *genotypes as a predictor of mutagen sensitivity have not been previously studied. Although we found no overall association for the *BRCA1 *E1038G, D693N, and Q356R genotypes with MBPC, in a polygenic model, the 1038G and 356Q *BRCA1 *SNPs predicted higher MBPC. These SNPs, however, have had only limited study for breast cancer risk, and null results were reported for women from high risk families [16-20] and sporadic breast cancer [21-23] possibly because of their low minor allele frequencies in the general population. The Q356R SNP has been studied, but both positive association and null results have been reported in sporadic breast cancer [21-23], and a positive association reported in one study of familial breast cancer [17], but not in another [16]. For the Q356R and E1038G SNPs, *in silico *analysis indicated that these could have adverse effects due to their location in the *BRCA1 *gene [25]. Recently, the 1038G polymorphism, which was in LD with 1183R, 871L, and 1613G in our study set, as well as in the HapMap CEU data (Utah residents with ancestry from northern and western Europe), was associated with increased BRCA1 protein expression in a small case-control study of breast cancer risk [66]. However, the same study did not find an association with K1183R, P871L, and S1613S, indicating that population admixture may have contributed to differences in haplotype frequencies.

The *BRCA1 *haplotypes examined herein were constructed from the 35 sequenced subjects and HapMap data, using the E1038G SNP, Q356R and D693N SNPs. Given the presumed detrimental effects of these SNPs on *BRCA1 *based on the *in silico *analysis [25], it would seem that the 3 SNP haplotype, CTC, would be associated with increased MBPC in our study. To the contrary, we found that the CTC haplotype was significantly associated with decreased MBPC. This association, though, is based on simulated data that only found significant associations in 4 of 10 simulations, using a frequency >3. Given that this haplotype is made up of the minor alleles of our SNPs, it is rare. Other studies evaluating BRCA1 haplotypes have used a combination of the SNPs used in our study to evaluate risk in sporadic breast cancer [22] and interactions with hormonal therapy [21], but we are the first to have used the 3-SNP haplotype found to be associated with low MBPC in our population. For example, Freedman and coworkers genotyped 28 BRCA1 SNPs, including the E1038G and Q356R SNPs, and observed 13 common haplotypes, but, none were associated with risk [22]. The Marie-Genica group studied a haplotype consisting of the Q356R (rs1799950), P871L (rs799917), and K1183R (rs16942) SNPs and found that carriers of one haplotype were at a higher risk of developing breast cancer after estrogen therapy use compared to those with the common haplotype [21]. Although the results are mixed for these genotypes and haplotypes for breast cancer risk, the genotype-phenotype associations indicate that further study is warranted.

For *Rad51*, sequencing was completed for 92 women, namely those with the highest and lowest MBPC. In this study, the *Rad51 5'UTR 135G→C *was found to be associated with decreased DNA repair capacity among unrelated subjects, in agreement with other findings [42,67]. Several *Rad51 *variants were identified and confirmed by reverse sequencing. However, consistent with the NCBI databases (http://www.ncbi.nlm.nih.gov/sites/entrez), these were found in very low frequency (<1%), with the exception of the *Rad51 5'UTR 135G→C*. According to HapMap, *Rad51 *has only a single haplotype block and SNP frequencies are low (<10%). Thus, haplotypes for *Rad51 *were not studied. For this DNA repair gene, the lack of observed genetic variation in functional components of the gene and homology across species [45] indicate that genetic variation in this gene might have detrimental effects. The 5'UTR 135G>C SNP, studied herein, can affect mRNA stability and/or translation efficiency, leading to altered product levels [41,42]. One study examined the effects of a *Rad51 *genotypes in *BRCA1/2 *carriers and reported that *Rad51 *135G>C genotype association with breast cancer risk was greater in *BRCA1 *carriers with truncating mutations (i.e. 185delAG) [40].

The *Rad51 *5'UTR variant C allele was then tested in the WEB case-control study because of the *a priori *hypotheses developed from the MSA assays. For the case-control analysis, we used the most parsimonious model because none of the other covariates, such as education, body mass index, age at first birth, age at menarche, age at menopause (for post-menopausal women only), number of births, and previous benign breast disease, changed the impact the genotype has on the odds of disease by greater than 5%. Including non-genetic variables that do not affect the impact of the genotype on odds of disease may actually mute the effect. Our results, however, did not indicate that the SNP was associated with breast cancer risk as a main effect, which is consistent with other studies [39,68].

The strength of this study lies using MBPC, a validated intermediate phenotype for breast cancer, as the dependent variable in EBV-immortalized cell lines from a large number of *BRCA1 *mutation carriers in order to assess genotype-phenotype associations. The advantage of evaluating an intermediate phenotype rather than the actual clinical phenotype, such as disease, is that the number of genetic and environmental factors influencing the intermediate is probably smaller than the number of factors affecting the disease resulting in a better powered study. Because the clinical outcome is taken out of the equation, the risks of spurious associations are limited and in fact, we can better explore the mechanisms of the disease. And, while this study assessed the effect of *BRCA1 *and *Rad51 *genetic variation on MBPC and risk of sporadic breast cancer, other genes in the HR pathway, such as BRCA2, PALB2, MERIT40, and others, could potentially be assessed for effects on the breast cancer intermediate phenotype, DNA repair capacity, as well as risk.

The use of *a priori *hypotheses also helped identify the most plausible SNPs to be examined in our family- and population-based epidemiological studies. Furthermore, the present study used data from a large case-control study of environmental exposures in the etiology of sporadic breast cancer. These types of studies can provide corroborative evidence to epidemiological studies of breast cancer.

This study does have some limiting factors. The FCR study subjects were small in number, limiting statistical power for detecting genotype-phenotype relationships. Additionally, while removing BRCA1/2 mutation carriers that had received prophylactic mastectomy and oophorectomy was reasonable, it is also possible that this may have introduced selection bias and that the reason that some women chose prophylactic surgery may have been because their perceived risk was greater, perhaps due to higher family penetrance. If *Rad51 *variants were an underlying factor for the increased penetrance and the subjects were excluded from the present study, it is possible that these exclusion criteria would decrease power.

The MBPCs in this study were lower than in other studies [6-8,69-71]. However, this difference may be due to the lower radiation dose and lower post-radiation incubation time used in this study. Although lower, the dose of 1 Gy for γ-irradiation and post-irradiation incubation time (4 hours vs. 0.5-1.5 hours) [6-8,69-71] conditions were chosen herein based on experiments identifying the optimal cell survival at the highest dose for these cell lines; dose-response evaluations showed 100% cell death at 2 Gy (data not shown). Radio-sensitivity due to germ-line *BRCA1 *mutations could also result in a lower dose response. Another explanation for lower MBPCs could be that only frank chromatid breaks were counted and all gaps were excluded, whereas other studies counted gaps as well. Ultimately, several associations were found making it 1) important to explore these associations among women without BRCA1 mutations and 2) essential to replicate in our larger case-control study.

## Conclusions

In conclusion, this study found an association for the MSA and breast cancer risk using subjects from a familial breast cancer registry. The MSA was then used as a phenotype to identify associated genetic variants, and several were found. These SNPs, namely the *Rad51 *5'UTR 135 C>G, *BRCA1 *E1038G and *BRCA1 *Q356R *BRCA1 *genotypes, and one *BRCA1 *haplotypes might be risk modifiers for *BRCA1 *mutation carriers. Our results provide evidence that deficient DNA repair may be a biomarker to identify higher-risk individuals in *BRCA1 *families, and provides an *a priori *hypothesis for further studies of *BRCA1 *and *RAD51 *SNPs in familial breast cancer risk.

## Competing interests

The authors declare that they have no competing interests.

## Authors' contributions

LJR-S conceived the study, carried out the molecular genetic studies, and drafted the manuscript. LES participated in the statistical design of the family study and carried out the statistical analysis of the family data. YY participated in the statistical design of the study and carried out the statistical analysis of the family data. JLF participated in the design of the study and coordinated the WEB study. CJI participated in the design of the family study, provided subject epidemiologic data, and coordinated the statistical analysis of the family data. MDS participated in the design of the family study, provided subject epidemiologic data, and helped carry out mutation analyses of BRCA1 and BRCA2 in the subjects. RGD carried out the mutagen sensitivity assay. CM carried out the haplotype analysis. JN carried out the final statistical analysis of the WEB study. DV participated in the design of the study and coordinated the WEB study. SBE participated in the design of the study and coordinated the WEB study. PGS conceived the study and participated in the design of the study. All authors read and approved the manuscript.

## Pre-publication history

The pre-publication history for this paper can be accessed here:

http://www.biomedcentral.com/1471-2407/11/278/prepub

## Supplementary Material

Additional file 1 **Summary table of MBPC in unaffected compared to affected subjects in all subjects and unrelated subjects**. This is a description of the MBPC in Unaffecteds and Affecteds in related and unrelated individuals. Click here for file

Additional file 2 **Characteristics of Study Sample by Case-Control Status and *Rad51 *Genotypes**. This table describes the characteristics of the WEB study participants by case-control status and genotypes. Click here for file
