# Supplementary material for: Association of Rad51 polymorphism with DNA repair in BRCA1 mutation carriers and sporadic breast cancer risk
Source: BMC Cancer. 2011 Jun 27;11:278. doi: 10.1186/1471-2407-11-278 (PMC3146938; doi:10.1186/1471-2407-11-278)
Supplement: Additional file 1 — Summary table of MBPC in unaffected compared to affected subjects in all subjects and unrelated subjects. This is a description of the MBPC in Unaffecteds and Affecteds in related and unrelated individuals. [file 1471-2407-11-278-S1.DOC]

Additional file 1

Title: **Summary table of MBPC in unaffected compared to affected subjects in all subjects and unrelated subjects.**

Description: This is a description of the MBPC in Unaffecteds and Affecteds in related and unrelated individuals.
