# Supplementary material for: Association of Rad51 polymorphism with DNA repair in BRCA1 mutation carriers and sporadic breast cancer risk
Source: BMC Cancer. 2011 Jun 27;11:278. doi: 10.1186/1471-2407-11-278 (PMC3146938; doi:10.1186/1471-2407-11-278)
Supplement: Additional file 2 — Characteristics of Study Sample by Case-Control Status and Rad51 Genotypes. This table describes the characteristics of the WEB study participants by case-control status and genotypes. [file 1471-2407-11-278-S2.DOC]

Additional file 2

Title: **Characteristics of Study Sample by Case-Control Status and *Rad51* Genotypes.**

Description: This table describes the characteristics of the WEB study participants by case-control status and genotypes.
